# Magnetic Resonance Fingerprinting - a promising new approach to obtain standardized imaging biomarkers from MRI

**DOI:** 10.1007/s13244-015-0403-3

**Published:** 2015-03-24

**Authors:** European Society of Radiology (ESR)

**Affiliations:** Neutorgasse 9/2, 1010 Vienna, Austria

**Keywords:** Magnetic resonance (MR), Magnetic resonance fingerprinting, Quantitative MR imaging, Multiparametric MR imaging

## Abstract

Current routine MRI examinations rely on the acquisition of qualitative images that have a contrast “weighted” for a mixture of (magnetic) tissue properties. Recently, a novel approach was introduced, namely MR Fingerprinting (MRF) with a completely different approach to data acquisition, post-processing and visualization. Instead of using a repeated, serial acquisition of data for the characterization of individual parameters of interest, MRF uses a pseudo randomized acquisition that causes the signals from different tissues to have a unique signal evolution or ‘fingerprint’ that is simultaneously a function of the multiple material properties under investigation. The processing after acquisition involves a pattern recognition algorithm to match the fingerprints to a predefined dictionary of predicted signal evolutions. These can then be translated into quantitative maps of the magnetic parameters of interest. MR Fingerprinting (MRF) is a technique that could theoretically be applied to most traditional qualitative MRI methods and replaces them with acquisition of truly quantitative tissue measures. MRF is, thereby, expected to be much more accurate and reproducible than traditional MRI and should improve multi-center studies and significantly reduce reader bias when diagnostic imaging is performed.

*Key Points*

• *MR fingerprinting* (*MRF*) *is a new approach to data acquisition*, *post*-*processing and visualization*.

• *MRF provides highly accurate quantitative maps of T1*, *T2*, *proton density*, *diffusion*.

• *MRF may offer multiparametric imaging with high reproducibility*, *and high potential for multicenter*/ *multivendor studies*.

## Overview

Magnetic resonance (MR) techniques such as MR spectroscopy and MRI are widely used throughout physics, biology and medicine because of their ability to generate detailed information about numerous important material or tissue properties, including those reflective of many common disease states [1, 2]. Tissues in the human body can be distinguished with magnetic resonance imaging (MRI) depending on their MR parameters, such as the longitudinal T1 relaxation, the transverse T2 relaxation, and the proton density (PD). In clinical routine, the MR system settings, such as echo time (TE), repetition time (TR), and flip angle, are most often chosen to highlight, or saturate, the image intensity of tissues, resulting in T1-weighting or T2-weighting in an image defined by such a contrast. There has therefore been a drive to quantitative MR imaging such as calculation of T1 and T2 relaxation times and ADC values from diffusion weighted imaging to develop imaging biomarkers that complement subjective radiological assessment [3]. However, the MR signal intensity is almost never quantitative by itself. The same material can have different intensities in different data sets depending on many factors, including the type and set-up of the scanner and coils used (B0 and B1 heterogeneities, RF pulse profiles), protocol related issues such as vulnerability to parameter changes, reconstruction issues such as noise floor, echo spacing etc., calibration issues (phantoms) and others. Because of this, in clinical MRI today, a tissue or material is typically referred to as being ‘hyperintense’ or ‘hypointense’ compared to another area, which may not provide a quantitative indication of the severity of the differences, and may have reduced sensitivity to global changes. A major disadvantage of using such comparisons is that the absolute intensity has no direct meaning and diagnosis relies on comparison with surrounding tissues in the image. Quantitative analysis of MR parameters on the other hand has so far been largely used to determine differences between spectral peaks, spatial locations or different points in time.

Thus robust, fully quantitative multi-parametric acquisition has long been the goal of research in MR. However, the quantitative methods developed to date typically provide information on a single parameter at a time, require a relatively long scan time, and are often highly sensitive to system characteristics. Simultaneous, multi-parametric measurements are almost always impractical owing to scan time limits and a high sensitivity to the measurement set-up and experimental conditions. Therefore the development of imaging biomarkers in MR and their widespread use in multi-centre studies has posed a big problem due to the enormous challenge of standardization in MR.

An even more definitive approach is the absolute quantification of the tissue parameters T1, T2, and PD. In this case, pathology can be examined on a pixel basis to establish the absolute deviation compared to the normal values. Automatic segmentation of such tissue images would be straightforward and the progress of the disease could then be expressed in absolute numbers. Although the advantages of absolute quantification are obvious, its clinical use is still limited. At least two major hurdles need to be addressed to stimulate widespread clinical use. For many methods, the excessive scan time associated with the measurement of the three parameters has so far prohibited its clinical application. However, in recent years there has been substantial progress for absolute quantification of T1, T2, PD of a whole volume with high resolution in a mere 5 min [4, 5]. The second hurdle, which must not be underestimated, is the clinical evaluation of the images. So far, there is only limited experience in using absolute T1, T2, and PD maps in clinical routines and most radiologists will want to confirm their findings using conventionally-weighted contrast images. The quantification scan might then be considered as superfluous in the limited time available for an examination. This item is addressed using the approach of synthetic MRI. It is possible to synthesize any T1-weighted or T2-weighted contrast image based on the absolute parameters, by calculating the expected image intensity as a function of a virtual set of scanner settings. Synthetic MRI can be seen as a translation of the absolute maps into conventional contrast images; thus, a single quantification scan can provide both the absolute maps and the contrast images for the examination.

Recently, a novel approach was introduced, namely MR Fingerprinting (MRF) [6] that may overcome these constraints by taking a completely different approach to data acquisition, post-processing and visualization. Instead of using a repeated, serial acquisition of data for the characterization of individual parameters of interest, MRF uses a pseudo randomized acquisition that causes the signals from different tissues to have a unique signal evolution or ‘fingerprint’ that is simultaneously a function of the multiple material properties under investigation. The processing after acquisition involves a pattern recognition algorithm to match the fingerprints to a predefined dictionary of predicted signal evolutions. These can then be translated into quantitative maps of the magnetic parameters of interest.

MRF is related to the concept of compressed sensing [7], and shares many of its predicted benefits. For example, preliminary results show that MRF could acquire fully quantitative results in a time comparable to a traditional qualitative MR scan, without the high sensitivity to measurement errors found in many other fast methods. Most importantly, MRF has the potential to quantitatively examine many MR parameters simultaneously given enough scan time, whereas current MR techniques can only examine a limited set of parameters at once [8]. Thus MRF opens the door to computer-assisted multi-parametric MR analyses, similar to genomic or proteomic analyses that could detect important but complex changes across a large number of MR parameters simultaneously. When an appropriate pattern recognition algorithm is used, MRF also provides a new and more robust behaviour in the presence of noise or other acquisition errors that may lead to the near complete suppression of deleterious effects stemming from these factors.

## MR fingerprinting method description

An inversion recovery based steady-state free precession MR sequence is proposed, since this sequence type is particularly sensitive to changes in T1, T2, and off-resonance frequencies and provides the highest signal-to-noise ratio [6, 9]. For signal readout, a fast sampling trajectory based on variable density spiral readout is applied and spatial undersampling used to speed-up measurement times. MRF acquisition patterns with randomized excitation flip angels, repetitions times, echo times, and inversion times, would fulfil the compressed sensing criteria for sparse reconstruction. Several slices (~10 slices) with 20 % slice gap and 3-5 mm slice thickness would cover the major pathological region and provide sufficient coverage representative for different regions. Automatic positioning would be performed by established scanner software (i.e., Auto align) to ensure comparable positing of slices for reproducibility measurements in healthy volunteers. Reproducibility scans can be performed consecutively in the same measurement session. The spatial resolution of the acquired images requires at least a 256×256 matrix with <1.3 mm isotropic resolution. The scan time requirements for MRF can be ~25 sec per acquired slice and an exact optimization of the parameter settings done at the outset. A total number of ~3000 excitations results in a total MRF scan time below 10 minutes.

In addition to MRF, conventional MR techniques during the study examination can be performed for three purposes: topographic comparison of MRF and traditional routine MR sequences and localization of the pathologic region.

These routine MR sequences will include short prescans including localizer sequences, Autoalign sequences for positioning, MR field map sequences for optimization of B0 field homogeneities, assessment of B1 homogeneity, and qualitative methods such as fluid-attenuated inversion recovery (FLAIR), T2-weighted turbo spin echo sequences, as well as established quantitative MR sequences for measurements of T1 and T2 such as multi-contrast spin echo based sequences and fast double inversion recovery based MR sequences [6, 8, 9]. The overall imaging time of the routine MR sequences should be below 30 min.

## Data processing

MRF data processing is performed online at the MR scanner based on a dedicated “fingerprinting dictionary” [6]. This dictionary was determined based on the MRF sequence setup (i.e., timing, flip angles etc.) and based on known development of spin evolutions during the MR sequence evolution using the Bloch equations. Matching of the measured data with the MRF dictionary is performed voxel-vise based on least squares correlation. The dictionary has to be setup to match the natural T1, T2, proton density, and off-resonance distributions that are routinely expected for in vivo MR scans at 1.5 T MR Scanners.

Meanwhile in addition to T1 and T2 relaxation times, proton density, and off-resonance distributions new reports demonstrate the possibility of acquiring diffusion property data and perfusion information by MRF [9, 10]. This opens the door for a completely new approach towards imaging biomarkers in many applications of MRI, such as neuro, oncology, musculoskeletal, cardiovascular, metabolism and chest.

## Conclusion

MRF could theoretically be applied to most traditional qualitative MRI methods and may replace them with acquisition of truly quantitative tissue measures. MRF is expected to be much more accurate and reproducible than traditional MRI and should improve multi-centre studies and significantly reduce reader bias when diagnostic imaging is performed. In addition, this new approach with MRF has potential to provide highly reproducible, accurate and fast quantitative imaging of several MR parameters simultaneously, and allow development of reliable and standardized imaging biomarkers and their multi parametric combinations, which can be validated in multicenter studies because of their good reproducibility across sites and even across different MR vendors.
